# Bicentre, randomized, parallel-arm, sham-controlled trial of transcranial direct-current stimulation (tDCS) in the treatment of palliative care patients with refractory cancer pain

**DOI:** 10.1186/s12904-023-01129-0

**Published:** 2023-02-28

**Authors:** Jean-Paul Nguyen, Hélène Gaillard, Alcira Suarez, Élie Terzidis-Mallat, Diane Constant-David, Aurélien Van Langhenhove, Adrien Evin, Catherine Malineau, Son V. O. Tan, Alaa Mhalla, Jean-Pascal Lefaucheur, Julien Nizard

**Affiliations:** 1 Unité de Stimulation Transcrânienne, Clinique Bretéché, Groupe Elsan, Nantes, 44000 France; 2 UIC22 et Service Douleur Soins Palliatifs et Soins de Support, Centre Hospitalo-Universitaire (CHU), Nantes, 44930 France; 3 Unité de Soins Continus, Clinique Bretéché, Groupe Elsan, Nantes, 44000 France; 4 Service de Neurochirurgie, University Hospital, Ho Chi Minh ville, Vietnam; 5 EA43910, Faculté de Médecine, Université Paris-Est, Créteil, 94000 France; 6 Unité Douleur et Soins Palliatifs intégrés, DMU Cancer et spécialités, CHU Henri Mondor-Albert Chenevrier, APHP, Créteil, 94000 France; 7 Unité de Neurophysiologie clinique, Hôpital Henri Mondor, APHP, Créteil, 94000 France

**Keywords:** tDCS, Palliative care, Cancer pain, Treatment, Randomized trial

## Abstract

**Background:**

Pain is a common symptom in palliative care cancer patients and is often insufficiently relieved. In recent years, transcranial direct-current stimulation (tDCS) of the motor cortex has been shown to be effective to treat chronic pain, essentially neuropathic pain. We propose to test the efficacy of tDCS in patients experiencing cancer pain in the palliative care setting.

**Method/design:**

This article describes the protocol of a bicentre, randomized, parallel-arm, sham-controlled clinical trial evaluating tDCS in the treatment of palliative care patients with refractory cancer pain. Seventy patients between the ages of 18 and 80 years experiencing refractory pain with a pain score of 4/10 on a numerical rating scale (NRS) ranging from 0 to 10 will be enrolled in this trial. The main exclusion criteria are patients unable to fill in the various rating scales and life expectancy less than 3 weeks. Treatment consists of 5 consecutive tDCS sessions targeting the motor cortex (one daily session for 5 days) on the contralateral side to the pain. After randomization (1:1 ratio), 35 patients will receive active stimulation and 35 patients will receive sham stimulation. The primary endpoint is the NRS score and the primary objective is a significant improvement of this score between the baseline score recorded between D-3 and D-1 and the score recorded 4 days after stopping treatment (D8). The secondary objectives are to evaluate whether this improvement is maintained 16 days after stopping treatment (D21) and whether the following scores are improved on D14 and D21: Brief Pain Inventory, Edmonton Symptom Assessment System, Hospital Anxiety and Depression scale, State-Trait Anxiety Inventory and Medication Quantification Scale.

**Discussion:**

Positive results of this trial would indicate that tDCS can improve pain and quality of life of cancer patients in the palliative care setting. Reduction of analgesic consumption and improvement of activities of daily living should allow many patients to return home with a decreased workload for caregivers.

## Background

Pain is a common symptom in palliative care cancer patients and is often insufficiently relieved [1, 2]. The 2010 INCA report showed that France is not an exception to this worldwide observation (synopsis of the 2010 national survey). This report shows that pain is the symptom that these patients fear the most and that it dramatically impacts their quality of life. These patients may experience so-called nociceptive pain related to stimulation of sensory nerve endings by the tumour. When tumour resection is impossible, a symptomatic analgesic treatment is generally proposed, mainly consisting of administration of opioid analgesics [3]. At high doses, this treatment induces adverse effects, especially drowsiness [4] and psychomotor retardation that impair the patient’s quality of life [5]. They may also experience so-called neuropathic pain, secondary to anatomical lesions or functional impairment of nerve structures (peripheral nerves or cerebral or spinal tracts) related to repeated surgical procedures and/or radiotherapy. This type of pain may respond to antiepileptic or antidepressant drugs [6]. At high doses, these treatments also induce adverse effects fairly similar to those observed during treatment of nociceptive pain [7]. As these two types of treatment often need to be coprescribed [8], these patients frequently present an almost permanent state of drowsiness at the end of life, preventing all normal activities of daily living [9–11].

In recent years, noninvasive brain stimulation (NIBS) techniques (transcranial magnetic stimulation (rTMS) or transcranial direct-current stimulation (tDCS)) have been successfully used to treat chronic pain [12]. We have shown that these NIBS techniques can improve pain in cancer patients in the palliative care setting [13–15]. In these case reports, which concerned patients at the end of life, analgesic drugs were able to be significantly decreased, allowing resumption of certain activities of daily living and a marked improvement of quality of life. Two of the 3 patients of these publications were able to return home. A publication by Knotkova [16] showed that tDCS also improved palliative chemotherapy-induced cognitive disorders, but these patients apparently did not experience any pain. Finally, two recent studies have confirmed the efficacy of NIBS in the treatment of cancer pain. Both of these studies concerned patients with visceral pain, as in two of our cases, but these patients were generally not at the end of life [17, 18]. In the study by Khedr, 17 patients were treated by 10 rTMS sessions targeted to the motor cortex and another 17 patients were treated by 10 sham rTMS sessions. These patients experienced visceral pain related to cancer progression, mainly involving the liver or pancreas. Only 2 patients were in the palliative care setting and died after 5 rTMS sessions. Evaluation was based on a pain visual analogue scale, a verbal scale and the Hamilton rating scale for depression [19]. All of these scores were significantly improved at the end of treatment (day 10) and this improvement persisted for 2 weeks, but was no longer observed at 1 month. In the study by Ibrahim et al. [18], 20 patients were treated by 10 tDCS sessions targeted to the motor cortex and another 20 patients were treated by 10 sham tDCS sessions. All patients experienced visceral pain related to progression of liver cancer. The rating scales were the same as those used in Khedr et al. study [17]. Actively stimulated patients were improved by the 5th session and remained improved for an average of 1 month.

tDCS appears to be more suitable than rTMS for the treatment of palliative care patients, who are often difficult to mobilize, as tDCS can be delivered at the patient’s bedside and possibly even at home, which is not the case with rTMS. tDCS also appears to be rapidly effective (after 5 sessions) in the context of cancer pain, and this effect lasts longer than that of rTMS.

The proposed treatment of refractory cancer pain by tDCS in palliative care patients is a new treatment modality that is well adapted to hospitalised patients. Each patient will receive 20 minutes of transcranial direct-current stimulation daily for 5 consecutive days. One arm will receive active stimulation and the control arm will receive sham stimulation. Patients and investigators will be blinded to the type of tDCS. By improving the patient’s activities of daily living, this treatment will enable the patient to return home under good conditions for both the patient and the caregivers. This treatment can also be continued at home. This strategy is consistent with current guidelines in this field, in which the priorities are improvement of quality of life [20–22], return home [23–25] and decreased workload for caregivers [26].

### Interventions: tDCS and follow-up intervention

tDCS was tested in healthy subjects from 2000 until 2005, at the time of the first clinical applications in the treatment of chronic pain. tDCS consists of delivering a low-intensity (1 to 2 mA) direct electrical current by means of a pair of electrodes (anode and cathode) applied to the scalp. Electrodes generally have a diameter (round electrode) or a diagonal (rectangular electrodes) ranging from 2 to 3.5 cm. To stimulate a given cortical zone, the anode is placed over of the selected zone, generally identified by means of an EEG headset (10/20 System Positioning). For the treatment of pain, the anode is placed over the primary motor cortex (M1) on the contralateral side to the pain or on the left side in patients with diffuse pain. The cathode is placed over a supposedly neutral cortical zone, usually the contralateral supraorbital cortex with respect to the anode. In this study, the stimulation intensity will be 2 mA using round sponge electrodes 3.5 cm in diameter. As in the case of rTMS, stimulation of M1 is thought to be active by means of the connections between the motor cortex and numerous structures situated away from M1 involved in pain modulation [27]: especially the cingulate cortex, thalamus and rostral ventromedial medulla [28, 29].

A tDCS session generally lasts 20 minutes. The patient may experience a feeling of heat or paraesthesia at the site of the electrodes for the first 30 seconds, but subsequently does not experience any sensations, which is why active stimulation will be delivered for the first 30 seconds of the sham procedure [30, 31]. In this study, patients will receive one daily session for 5 consecutive days. The HDC Kit (Newronika, Milano, Italy) will be used. The system includes a programming module, which allows to choose an active or placebo stimulation mode, which is independent of the stimulation module where there is no indication on the stimulation mode. In this protocol, a nurse programs the device according to the results of the randomization, then is no longer involved in the protocol. The physician who performs the stimulation, using the previously programmed stimulation module, the patient and the evaluating physicians are not aware of the programming mode, and are thus blind.

This study will be conducted in patients hospitalised in a unit experienced in palliative care. tDCS will be delivered daily for 5 consecutive days (from Monday to Friday) with the patient either sitting or lying down.

### Benefits and risks

At the individual level, pain intensity is expected to decrease by the 2nd or 3rd tDCS session, with a more marked improvement after the 5th session. This improvement is expected to last at least 4 days (D8) and up to 16 days (D21) [15, 18]. It should be possible to markedly decrease analgesic drug treatment during this period, which should result in less drowsiness and improved activities of daily living. An improvement of sleep, tiredness and mood can also be expected. The patient may be able to return home during the week after the end of treatment with a markedly reduced workload for the patient’s caregivers.

Side effects are recorded in the electronic observation booklet after each tDCS session (D0, D1, D2, D3, D4) and during the 3 subsequent evaluations (D8, D14 and D21). Side effects [32] such as headaches or vomiting that are repeated or even worsen with each tDCS may force the patient to stop the treatment if they are prolonged or poorly tolerated. The most frequent side effect is a bad tolerance of the stimulation itself. The patient may have a sensation of electrical discharge or burning sensations at the positioning of electrodes*,* especially in the event of excessive stimulation (> 2 mA) delivered by excessively small electrodes (< 20 × 20 mm) [32]. At the most, there may be a real burn on the scalp in contact with the electrode. Initially, the proper impregnation of the sponge electrodes by the saline solution will be checked and if the phenomenon continues, the stimulation intensity will be decreaed to 1.5 mA. In the event that these adverse effects persist, the treatment will be stopped. The risk of inducing seizures is almost non-existent, but it is recommended to avoid tDCS in patients with seizures not controlled by medical treatment.

A significant collective benefit could be observed, and tDCS could markedly decrease the length of hospital stay of these patients and their analgesic drug consumption and should therefore have a significant health economics impact. In parallel, the patient’s and caregivers’ quality of life could be significantly improved. In the longer term, continuation of tDCS at home would require a new organization. The development of remote treatment techniques must also be considered [24].

More generally, this protocol raises the issue of clinical research in palliative care patients [11], which has been the subject of several studies [33–35], showing that palliative care patients potentially at the end of life were generally in favour of scientific studies designed to improve the quality of life of future patients, even in the absence of a direct personal benefit.

In order not to penalize patients in the placebo group, it is planned to lift the blind at D21 and to offer these patients the active treatment outside the protocol. Patients who have been actively treated and whose pain has recurred after D21 will be offered a second treatment outside the protocol.

As tDCS is a noninvasive technique with few expected adverse effects, the benefit/risk balance is expected to be highly positive for the patient in this context of refractory cancer pain in the palliative care setting.

### Study objective

This study is designed to evaluate the analgesic efficacy of 5 consecutive tDCS sessions to the primary motor cortex in patients with cancer pain in the palliative care setting.

The primary endpoint is the score of the numerical rating scale (NRS), which measures pain intensity on a scale from 0 to 10 [36]. The mean of 3 NRS scores recorded during the day (morning, midday and evening) will be used. The primary endpoint for analysis will be the mean variation of the pain NRS between the baseline assessment (D0) and the D8 assessment.

The secondary objectives will be to evaluate:

- Immediate impact of each tDCS session on pain intensity.
- Response rate at the end of treatment.
- Residual analgesic effect.
- Effects of TDCS on the other main symptoms likely to impair quality of life.
- Analgesic consumption on D0 and D8.

Secondary endpoints will be:

- Pain NRS score, MQS (Medication Quantification Scale [37]), including evaluation of opioid consumption expressed in oral morphine equivalent per day on D0 and D8.
- Pain NRS scores, recorded before and after each session.
- Efficacy of treatment, defined by a ≥ 20% reduction of the mean NRS score between D0 and D8 (IMMPACT recommendation in chronic pain [38]).
- Mean NRS score on D14 and D21 in the 2 arms.
- Completion of the following questionnaires and scales on D0 and D8:
  - . BPI (Brief Pain Inventory, short form [39]).
  - . ESAS (Edmonton Symptom Assessment System) [40].
  - . HADS (Hospital Anxiety and Depression Scale) [41].
  - . STAI-Y (State-Trait Anxiety Inventory (Form Y) [42]).
- Analgesic consumption assessed by MQS on D0 and D8.

Analysis of repeated measurements of quantitative secondary endpoints (NRS, BPI, ESAS, HADS, STAI-Y and MQS) will be based on the mean variations of the endpoint between the baseline assessment (D0) and the D21 assessment.

### General study methodology

This is a French bicentre (Clinique Bretéché and CHU/University Hospital Nantes), randomized, comparative, double-blind, sham-controlled trial conducted in two parallel arms: tDCS (35 patients) and sham tDCS (35 patients).

Centralized randomization will be performed by software and will be specific to each of the 2 centres. Patients will not be informed about whether they have been allocated to the active tDCS arm or the sham tDCS arm. Physicians performing the tDCS technique and the evaluating physicians will also be blinded to the type of treatment delivered (see “Interventions: tDCS and follow-up intervention”).

Doses of analgesic medications (opioids, antiepileptics and antidepressants) must be stable for at least 48 hours (D-3, D0) prior to inclusion in the study. The level of analgesic treatment will be assessed by MQS. Analgesic doses may be able to be decreased from the first day of tDCS depending on its analgesic effect. One of the objectives of tDCS is to decrease the doses of analgesics in order to avoid a state of drowsiness or torpor associated with impaired quality of life.

#### Inclusion criteria

- Patients between the ages of 18 to 80 years with cancer pain refractory to medical treatment (mean NRS ≥ 4/10 on 2 consecutive days) in the palliative care setting.
- Patients agreeing to participate in a research protocol in this palliative care setting.

#### Exclusion criteria

- Patients younger than 18 years or older than 80 years.
- NRS < 4/10 (pain controlled by adapted medical treatment and global management).
- Patients unable to fill in the various questionnaires, specifically chosen to be simple and easy to fill in.
- Patients refusing to sign the informed consent form.
- Patients with cognitive impairment, metal implantation in the brain and mood disorders
- Patients with a life expectancy less than 3 weeks (duration of study follow-up).

### Recruitment modalities

Patients will be recruited from the Center 1 (Clinique Bretéché rehabilitation and palliative care unit, Nantes), and Center 2 (CHU/University Hospital Nantes palliative care unit). The inclusion of one patient per month corresponds to the recruitment, treatment and follow-up capacities of each of these centres in accordance with the protocol. The study will last 24 months.

### Study plan (Fig. 1)

#### Screening visit (D-3)

Verification of inclusion and exclusion criteria. Patients will be provided with the information sheet and will be asked to sign the informed consent form. The patient will be provided with NRS forms (3 assessments per day for 48 hours). For organizational reasons, this screening visit will be held on a Friday. NRS scores will be recorded on Saturday and Sunday so that inclusion or exclusion of the patient can be decided on Monday.

**Fig. 1:**
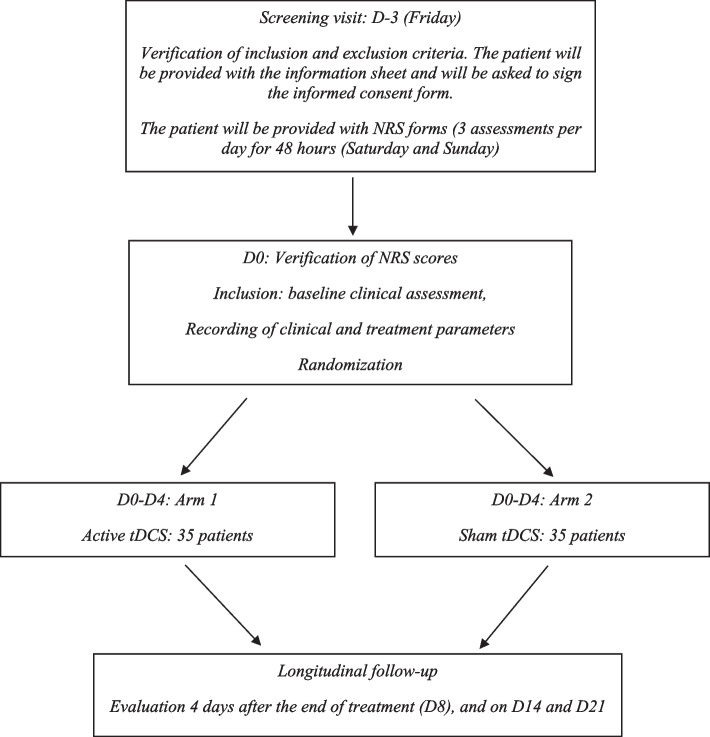
Study plan: the study will last 24 days (D-3 to D21) including a 3-day inclusion period (D-3-D0), a 5-day treatment period (D0-D4) and a 16-day follow-up period after stopping treatment (D8, D14, D21). Treatment consists of a daily tDCS session on 5 consecutive days. Patients will be randomized to 2 arms (1:1 ratio): active tDCS and sham tDCS. Patients and investigators will be blinded to the results of randomization (double-blind). The primary objective will be a significant reduction of pain evaluated by a numerical rating scale (NRS) from 0 to 10 between the baseline score recorded between D-3 and D0 and the score recorded 4 days after stopping treatment (D8)

#### Inclusion visit (visit 1) (D0)

Recording of NRS scores for 48 hours (D-3 to D-1) allowing inclusion of the patient. Recording of clinical parameters such as age, sex, weight, height, etc., medical history, previous and concomitant treatments, vital signs. Baseline assessment: NRS, BPI, ESAS, HADS, STAI-Y and MQS (see secondary endpoints). Randomization (by centre, 1:1 ratio). Study personnel (physician and nurses specialized in pain management) will be blinded to the results of randomization.

#### Treatment with active or sham tDCS (D0-D4)

Study personnel (physician or trained nurse), but not the patient, will be informed about the results of randomization. tDCS will be delivered daily for 5 consecutive days. The NRS will be scored 3 times a day on these 5 days from Monday to Friday.

#### Visit 2 (D8)

Review of adverse events (AEs), recording of NRS scores and the various other assessment scores (BPI, ESAS, HADS, STAI-Y and MQS) 4 days after stopping treatment.

#### Visit 3 (D14, 9 days after stopping treatment) and visit 4 (D21, 16 days after stopping treatment)

The same parameters as those assessed on D8 will be assessed at these visits. These parameters will reflect the patient’s state 9 days and 16 days after stopping treatment. Patients who have returned home in the meantime will be assessed at home. In every case, the assessment will be performed by a team blinded to randomization Table 1.

**Table 1:** Study calendar

Actions	Screening visit (D-3)	D0	D0-D4	D8	D14	End of study visit (D21)
Informed consent, demographic and clinical data	X					
Clinical examination	X	X	X	X	X	X
Randomization		X				
NRS	X	X	X	X	X	X
tDCS			X			
Recording of AEs			X	X	X	X
BPI, ESAS, HADS, STAI-Y, MQS		X		X	X	X

*NRS* Numerical Rating Scale, *AEs* adverse events, *BPI* Brief Pain Inventory, *ESAS* Edmonton Symptom Assessment System, *HADS* Hospital Anxiety and Depression scale, *STAI-Y* State-Trait Anxiety Inventory (Form Y), *MQS* Medication Quantification Scale

##### Patient follow-up

In order to maximize the number of patients evaluable for the primary analysis on the mITT population (modified intention-to-treat [43]), in the event of treatment discontinuation during the D0-D4, patients will be maintained in the trial and evaluated according to the protocol at the D14 and D21 visits. Coordination between the 2 centers is ensured by a project leader (Mrs. Marine Royer) from ELSAN, who promotes the project, in conjunction with the clinical research nurse of each of the two centers. A videoconference meeting is organized every 2 months to review the progress of the protocol in each center.

### Clinical parameters

- The pain numerical rating scale (NRS) is a pain self-assessment tool. The patient rates his or her pain orally on a scale from 0 to 10, where 0 corresponds to the lower limit defining absence of pain and 10 corresponds to the upper limit defining the worst imaginable pain. The NRS was preferred to a VAS (visual analogue scale) based on the 2009 expert consensus on cancer pain assessment [44].

This scale can be used to include patients considered to experience sufficiently severe pain to justify more or less invasive treatment. For example, pain scored as 3/10 corresponds to moderate pain [45, 46], which should be controlled by global management and/or the use of mild analgesics (WHO step 1 [47]). Pain scored between 7 and 10/10 is considered to be major pain (between severe and maximum) [45]. Pain scored as 4/10 is considered to be moderate, but with a significant impact on the patient’s quality of life [48] and needs to be treated by noninvasive or minimally invasive modalities (WHO step 2 or 3 analgesics or noninvasive brain stimulation techniques, including tDCS). Invasive treatments (e.g. cervical cordotomy [49] or implantable pumps for intrathecal drug administration [50]) specifically intended for palliative care patients can be considered in patients experiencing pain with NRS scores higher than 6–7/10 and could be proposed in the event of failure of tDCS in the context of this protocol.

The validity and reliability of the NRS for the evaluation of the efficacy of analgesic treatment has been established [45]. A 20 to 30% improvement of the NRS score is generally considered to be significant [51]. This percentage improvement appears to be sufficient to assess the efficacy of a drug treatment associated with few adverse effects [52]. A higher level of efficacy (≥ 40% improvement) is generally required for surgical treatment in view of the higher risks involved. Various authors have considered that a noninvasive interventional treatment, such as tDCS, can be evaluated according to the same criteria as drug treatments [53, 54]. In the more specific field of palliative care, a 2-point improvement on NRS was estimated to be significant in 74.3% of cases in a group of 70 patients treated with ketamine infusion [55]. In 2 other reported cases, again in the palliative care setting, ketamine infusion resulted in a 2 and 3 point decrease in NRS, respectively, accompanied by clinical improvement deemed sufficient to allow the 2 patients to return home [56]. These results were the basis for our study’s enrollment calculations. Also in palliative care, it has been established that an assessment of pain, using only a scale such as the NRS was insufficient [57]. For this reason, the following scales were added to the study of clinical parameters.

- The Brief Pain Inventory (BPI) is a self-administered questionnaire constructed and tested in cancer pain [39]. It explores the main dimensions of pain: site, intensity, functional impairment, social repercussion, interpersonal relations and psychological distress. It will be used in this trial, as in most clinical trials.

The short form, composed of 9 items, has been validated and shown to be reliable in the assessment of cancer pain [38]. The BPI and its short version were validated in French in 2007 [58]. In palliative care, the BPI has been recommended for the assessment of adult patients without cognitive impairment [57]. It has been reported that some patients may have difficulty performing this assessment alone [59]. Our protocol concerns hospitalized patients who will all be assisted to complete the questionnaires.

- Evaluation of the various symptoms observed in the context of palliative care constitutes an evaluation of the quality-of-life approach in this setting. The Edmonton Symptom Assessment System (ESAS) has been validated and found to be one of the most reliable of the various scales [40]. This scale is relatively easy to score by drowsy and/or tired patients, in contrast with questionnaires like the McGill Quality of Life Questionnaire [60] or VOICES-SF [22]. ESAS evaluates 9 symptoms by means of a visual analogue scale (0–100 mm): pain, tiredness, nausea, depression, anxiety, drowsiness, lack of appetite, shortness of breath and wellbeing. In general, mean scores between 10 and 30, 40 and 60, and 70 and 100 mm reflect slight, moderate and severe discomfort, respectively [61].
- Since 1983, anxiety and depression are generally evaluated by the HADS (Hospital Anxiety and Depression Scale [41]). The HADS has been validated and recommended to evaluate these symptoms in the context of cancer pain and palliative care [62].
- The STAI-Y [42] is a self-administered questionnaire designed to study the state of anxiety experienced at a given point in time in adults and is composed of 20 questions, scored from 1 to 4.

Scores range from 20 to 80 and are classified in the following way:

- very high > 65,
- high: 56 to 65,
- moderate: 46 to 55,
- low: 36 to 45,
- very low ≤35.

To date, this scale has not been validated for the evaluation of cancer pain, nor in the context of palliative care. As much as an improvement of a possible depressive state could be explained by the action of the tDCS [63], it is not at all known that the tDCS can have a proper effect on anxiety. Anxiety may, however, contribute to the distress that can be encountered in a palliative care setting. A recent publication suggests that tDCS may have an effect on this state of distress [64], which highlights the interest of an own assessment of anxiety.

- The Medication Quantification Scale (MQS) is a scale used to quantitatively evaluate changes in analgesic drug administration [37]. Each medication is attributed a score corresponding to a class of drugs, mainly taking their adverse effects into account. Daily doses are also classified as a function of recommended dosages. The total score is the product of the score corresponding to the drug class and the score corresponding to its daily dose. This score has been used in 3 studies in palliative care patients treated by noninvasive transcranial brain stimulation techniques [13–15]. In this context, it proved to be reliable, its evolution reflecting well the clinical evolution of the 3 treated patients.

### Statistical analysis

Statistical justification of the sample size: a 2-point improvement of the numerical rating scale is considered to be clinically relevant [51], which is supported by data from the study conducted by Cheung [55] on the efficacy of ketamine in a population of patients in palliative care setting (see “Clinical Parameters”).

As this is the first protocol dedicated to the treatment of painful patients in a palliative care setting with tDCS, we did not find any evidence in the literature that could help us justify the number of needed patients for the evaluation of the other clinical parameters, apart from the NRS.

On the basis of these hypotheses:

- A mean NRS difference of 2 points between the active tDCS arm and the sham tDCS arm,
- Standard deviation of 2.8,
- Power of 80%.
- Alpha risk of 5%,

32 patients per arm would be necessary, i.e. a total of 64 patients. In order to ensure sufficient power, an additional 10% of patients will be included in the study, i.e. a total of 70 patients.

The study will therefore include 70 patients: 35 in the tDCS arm and 35 in the sham tDCS arm.

The variables measured on inclusion will be described for all patients and in each of the two arms by number and percentage for each modality for qualitative variables and by minimum, maximum, mean, standard deviation and quartiles for quantitative variables. The primary efficacy population will be a modified intention-to treat (mITT) population, comprising all randomized patients who have received at least one tDCS session (active or sham) and who were evaluated on D0 (baseline) and on D8.

#### Analysis of the primary endpoint

Analysis of the primary endpoint, the pain NRS, will be performed on the mITT population.

The mean NRS score on D8 will be compared between the active tDCS arm and the sham tDCS arm using a mixed linear model in order to take stratification factors into account.

#### Analysis of secondary endpoints

The mean NRS score on D8 will be compared between the active tDCS arm and the sham tDCS arm using a mixed linear model in order to take stratification factors into account. This analysis will also be adjusted for analgesic consumption based on the MQS score.

The immediate efficacy of tDCS on pain intensity will be compared between the two arms using a mixed linear model with the pre- and post-tDCS difference of the NRS score as the variable to be explained. This model will take repeated measures per patient into account.

The response rate will be compared between the two arms by a mixed logistic model in order to take stratification factors into account.

The residual analgesic effect on D14 and D21 will be studied using a mixed linear model in order to take stratification factors into account. This analysis will also be adjusted for analgesic consumption.

The course of quality of life assessed by the BPI, ESAS, HADS and STAI-Y between D0 and D7 will be compared between the two arms by a mixed linear model in order to take stratification factors into account.

Analgesic consumption, evaluated by the MQS questionnaire, will be compared between the two arms using a mixed linear model.

Complementary analysis of the primary efficacy endpoint could be conducted on a per protocol population including all patients of the mITT population not presenting any major protocol violations. These major violations will be defined in the analysis plan before locking the database.

### Randomization

Centralized randomization will be performed on D0 after verification of the inclusion criteria. Randomization will be performed under double-blind conditions and will be stratified according to the mean baseline pain intensity (4–6 or 7) over the last 48 hours before inclusion and according to centre.

Patients will be randomized to receive either active tDCS or sham tDCS for the 5 treatment days defined by the protocol. The randomization ratio will be 1:1. Randomization will be stratified by centre by means of the Ennov Clinical electronic CRF (https://fr.ennov.com/getions-essais-cliniques/) by logging onto the website: https://nantes-lrsy.hugo-online.fr/CSOnline/ after obtaining the patient’s informed consent on D0. The inclusion number and randomization arm will be automatically allocated at the time of randomization. A confirmation e-mail will be sent to the person who performed randomization and to all other people concerned.

Randomization lists will be established by a Nantes university hospital research promotion department statistician.

A patient registered in the trial, to whom treatment has been allocated and documented, will be considered to be randomized. A patient cannot be included, evaluated or randomized in the trial more than once.

## Discussion

The effect of motor cortex tDCS on cancer pain and its duration of action are important parameters that need to be assessed in order to define the optimal modalities of application of tDCS in the palliative care setting.

Up until now, the principle of neurostimulation techniques has been mainly based on the Gate Control theory elaborated by Melzack and Wall [65] to treat neuropathic pain. This principle was the basis for the development of percutaneous stimulation, thalamic stimulation and spinal cord stimulation [66–68]. These techniques have been demonstrated to be effective in the treatment of neuropathic pain, but they have no significant effect on nociceptive pain, essentially cancer pain. Invasive [69, 70] or noninvasive [27] motor cortex stimulation, which has been developed more recently, has also been shown to be effective on neuropathic pain [71]. It was rapidly demonstrated that noninvasive stimulation techniques (rTMS and tDCS) need to be repeated in order to ensure long-term maintenance of the clinical result [72, 73]. Some studies [27] have suggested that the analgesic effect of motor cortex stimulation is mainly due to stimulation of its connections with several structures situated away from the motor cortex, involved in modulation of pain in general, including nociceptive pain (cancer or non-cancer pain), especially the cingulate cortex, periaqueductal gray, and rostral ventromedial medulla [28, 29, 74, 75]. This mechanism could explain the efficacy of tDCS in cancer pain in the palliative care setting, in which nociceptive pain and neuropathic pain are often associated. The implication of the cingulate cortex [74] explains why the affective component of pain could be particularly improved. Similarly, activation of connections between the motor cortex and the dorsolateral prefrontal cortex could also be responsible for improvement of mood, which would be a useful effect in this context, in which a high percentage of patients are depressed [76]. The expected indirect effects of tDCS include a delayed but probably prolonged effect due to the plasticity phenomenon [27], generally induced by short-term repetition of tDCS sessions (one daily session for 5 days). In the series reported by Ibrahim [18], pain relief was especially marked after the 5th session and was maintained for an average of 1 month, as in our case [15]. This persistent effect of a protocol comprising 5 consecutive tDCS sessions has been observed in other clinical indications [77], which has justified the choice of this regimen for this protocol. In addition, choosing a more prolonged treatment, such as 10 consecutive days, would likely have penalized patients with sham tDCS, bearing in mind that some patients might have a short lifespan.

The origin of pain does not appear to affect the result. The main results concern visceral pain [17, 18], but one of our patients with pain related to vertebral metastases was also considerably improved [15]. In this palliative care setting, in which patients are often difficult to mobilize, tDCS is much easier to deliver than rTMS, as it can be performed at the patient’s bedside and possibly at home.

The other treatments that can be proposed instead of tDCS often have the disadvantage of being administered by infusion and/or of causing disorders of consciousness. Sedation is in some cases a desired effect [78], but it degrades the quality of life of the patient and his relatives, and is almost impossible to continue at home for very long. Ketamine is easier to handle and can eventually be given orally as a maintenance treatment [55, 79]. However, it remains overall difficult to maintain at home and is not without more serious and frequent side effects than with tDCS. A comparative study would be interesting.

One of the limitations of this protocol is the lack of a pilot study, which led us to make choices that were not always well documented in the development of the protocol. The first choice was to select the tDCS system best suited for a double-blind treatment protocol in a palliative care setting. There are now several devices available that meet these criteria well. The HDC Kit makes it easy to perform double-blind stimulation easily at the patient’s bed. The calculation of the number of patients could not be based on similar protocols. Those dedicated to cancer pain [17, 18] were aimed more at non-hospitalized patients with a priori a life span of more than 3 months. The only protocols performed in palliative care involved ketamine infusion treatments. Since we were dealing with the same population and in principle the same expectations (in particular the return home), we chose the same main criterion, which was NRS improved by at least 2 points. The date of the “end point” was also chosen in a way that is open to discussion. It would have been possible to choose D4 which corresponds to the date of the 5th day of treatment. This date also corresponds to the date of maximum improvement in a case we have previously treated [15]. However, there is almost always a post-effect that should allow patients to be stimulated sequentially, for example 5 days in a row every 10 or 15 days, which would be easier to perform in routine. Therefore, it was the evaluation of the quality and duration of the aftereffect that led us to choose D8 as the “end point” date.

We hope that this protocol will be able to confirm the value of tDCS in the treatment of cancer pain in palliative care patients, especially by facilitating a rapid return home.

## Data Availability

All data will be anonymized and stored under lock and key, with access granted to the research team only in each of the 2 centers.
